# Post-Translational Modifications of Proteins: Novel Insights in the Autoimmune Response in Rheumatoid Arthritis

**DOI:** 10.3390/cells8070657

**Published:** 2019-06-29

**Authors:** Francesco Carubbi, Alessia Alunno, Roberto Gerli, Roberto Giacomelli

**Affiliations:** 1 Rheumatology Unit, Department of Biotechnological and Applied Clinical Science, School of Medicine, University of L’Aquila, 67100 L’Aquila, Italy; 2 Department of Medicine, ASL1 Avezzano-Sulmona-L’Aquila, 67100 L’Aquila, Italy; 3 Rheumatology Unit, Department of Medicine, University of Perugia, 06129 Perugia, Italy

**Keywords:** rheumatoid arthritis, post-translational modifications (PTM) of proteins, antibodies against citrullinated proteins (ACPA), anticyclic citrullinated peptide antibodies (anti-CCP), antibodies against modified proteins (AMPA), biomarkers

## Abstract

Post-translational modifications (PTM) are chemical changes mostly catalyzed by enzymes that recognize specific target sequences in specific proteins. These modifications play a key role in regulating the folding of proteins, their targeting to specific subcellular compartments, their interaction with ligands or other proteins, and eventually their immunogenic properties. Citrullination is the best characterized PTM in the field of rheumatology, with antibodies anticyclic citrullinated peptides being the gold standard for the diagnosis of rheumatoid arthritis (RA). In recent years, growing evidence supports not only that a wide range of proteins are subject to citrullination and can trigger an autoimmune response in RA, but also that several other PTMs such as carbamylation and acetylation occur in patients with this disease. This induces a wide spectrum of autoantibodies, as biomarkers, with different sensitivity and specificity for diagnosis, which may be linked to peculiar clinical manifestations and/or response to treatment. The purpose of this review article is to critically summarize the available literature on antibodies against post-translationally modified proteins, in particular antibodies against citrullinated proteins (ACPA) and antibodies against modified proteins (AMPA), and outline their diagnostic and prognostic role to be implemented in clinical practice for RA patients.

## 1. Introduction

Rheumatoid arthritis (RA) is a chronic systemic inflammatory disease mainly affecting synovial joints [1]. In about 40% of patients, extra-articular manifestations also occur, thereby worsening disease prognosis and increasing mortality [2]. Auto-antibodies (auto-Abs) represent a hallmark of the disease, and the first auto-Ab linked with RA, the rheumatoid factor (RF), was described in the late 1940s [3]. RFs are auto-Abs against antigenic determinants on the Fc fragment of IgG molecules and include not only IgM but also IgG, IgA, and IgE RF variants [4]. A fairly recent meta-analysis showed that RF displays a sensitivity of 71% and a specificity of 83%, with a positive likelihood ratio (LR) of 3.96 for the diagnosis of RA [5]. Furthermore, the presence of IgA RFs in RA patients is associated with rapidly progressive, more severe disease and bone erosion, while both IgG and IgA RFs are associated with systemic manifestations [4].

A few years after the identification of RF, Nienhuis and Mandema reported that antibodies in sera from patients with RA reacted in a very specific way with keratohyalin granules present in buccal mucosa cells. A serological test based on this finding became known as the antiperinuclear factor (APF) test [6]. Almost 30 years later, Hoet and colleagues demonstrated that the perinuclear factor exactly colocalized with profilaggrin but failed to show that the antigen was identical to this protein [7]. Subsequently, the same investigators found that the antigen was not present in keratohyalin granules of cultured buccal mucosa cells, despite the presence of profilaggrin in these granules [8]. The evidence that filaggrin in fully differentiated buccal mucosa cells, in particular dead cells, is citrullinated, in contrast to profilaggrin in cultured living cells, is a milestone in the history of RA serology, marking the beginning of the era of antibodies against citrullinated proteins. Citrullination is one out of many post-translational modifications (PTMs) of proteins which are involved in physiological processes such as skin keratinization. A wide number of peptides could undergo PTMs, but the breakdown of tolerance to citrullinated peptides and eventually the development of auto-Abs against citrullinated proteins (ACPA) only occurs in predisposed individuals [9]. Furthermore, autoimmunity is only one consequence of increased citrullination, which can also lead to exposure of damage- and/or pathogen-associated molecular patterns (PAMP/DAMP) that can eventually be used by pattern recognition receptors (PRR) to provide entry and immune evasion [10]. Increased citrullination of proteins has been observed not only in RA but also in other inflammatory conditions, such as multiple sclerosis and atherosclerosis, but also in Alzheimer’s disease and tumorigenesis [11].

Nowadays, the term ‘citrullinome’ encompasses the wide range of citrullinated proteins identified by proteome analysis from biologic samples of RA patients [12]. In addition, antibodies against proteins that underwent PTMs other than citrullination in RA patients are defined antibodies against modified proteins (AMPA).

The need for biomarkers facilitating early diagnosis and profiling those individuals at the highest risk for a poor outcome has become of crucial interest for rheumatologists.

In this review, we provide an overview of the scientific literature about the diagnostic and prognostic role of ACPA and AMPA in RA. In particular, we designed a comprehensive literature search on this topic, by a review of reported published articles in indexed international journals up until 31st April 2019, following proposed guidelines for preparing a biomedical narrative review [13].

## 2. Antibodies against Citrullinated Proteins (ACPA)

Citrullination is a PTM catalyzed by the peptidylarginine deiminase (PAD) enzyme and inducing the conversion of arginine residues into the nonstandard aminoacid citrulline. To date, five isoforms of PAD have been identified. PAD1 and 3 are mainly expressed in the skin, PAD2 in the central nervous system and hematopoietic cells, and PAD4 and 6 in hematopoietic cells [14]. Citrullination causes the loss of a positive charge with a small change in the protein molecular mass, influencing the ability to form a hydrogen bond and ultimately the interaction with other aminoacid residues. Therefore, a citrullinated protein is fairly different from the original form, from a conformational and functional point of view. Although citrullination normally occurs in physiological processes, including skin keratinization, an increased expression of PAD genes on a genetic basis [15] and the creation of new immunogenic epitopes can induce an abnormal immune response in predisposed individuals [16]. ACPA are highly cross-reactive and bind a wide variety of citrullinated proteins, including citrullinated versions of enolase, fibrinogen, vimentin, collagen, and histones, among others [17]. Several studies identified such citrullinated proteins in RA synovial tissue, for example, citrullinated fibrinogen/fibrin [18] and citrullinated histones [19]. In addition, it is interesting to note that citrullinated fibrin is also present in the synovial tissue of patients with spondyloarthritis and psoriatic arthritis. This finding further reinforces the concept that protein citrullination is not specific of RA and does not necessarily leads to the development of ACPA [20].

Citrullination can be induced by several stimuli, such as cigarette smoking and periodontitis caused, in particular, by Porphyromonas gingivalis [14], the only known prokaryote expressing the PAD enzyme [21].

### 2.1. Predictive and Diagnostic Role of ACPA

Sensitivity and specificity are important measures of the diagnostic accuracy of a test. Sensitivity is the ability of a test to correctly identify patients with the disease, while specificity is the ability of a test to correctly identify subjects without the disease. Sensitivity and specificity are inversely proportional, meaning that it is up to the operator to decide whether a high sensitivity or a high specificity is more suitable according to the purpose of the test. A highly sensitive test, if negative, rules out the disease, while a highly specific test, if positive, confirms the suspicion of the disease. The gold standard is the best single test (or a combination of tests) that is considered the current preferred method of diagnosing a particular disease. Any new diagnostic test needs to be compared to the relevant ′gold′ standard [22,23].

Citrullinated filaggrin was the first citrullinated protein identified in RA, and the first tests for the presence of these antibodies employed either linear citrullinated peptides or citrullinated filaggrin as an antigen. Although they showed excellent specificity, the sensitivities obtained were, in most cases, relatively low [24]. To increase the sensitivity of the citrulline-containing peptide ELISA tests, the peptides were made cyclic to adopt a structure in which the citrullinated epitope is more efficiently recognized by the patient’s antibodies, with a sensitivity and specificity of 68% and 98%, respectively [25]. The first-generation anticyclic citrullinated peptide (CCP1) ELISA test used a filaggrin-derived cyclic peptide as the antigenic substrate. Subsequently, a peptide library screening of about 12 million peptides from dedicated synthetic peptide libraries was screened with RA sera, and the best citrullinated peptides were incorporated into a second-generation CCP test (CCP2). This test became commercially available in 2002 and is still recognized as the gold standard of testing for a type of ACPA, also called anti-CCP, in routine clinical practice [14]. The diagnostic relevance of anti-CCP is outlined by their inclusion, along with RF, in the 2010 American College of Rheumatology classification criteria for RA [26]. Importantly, not only their positivity but also their titer is taken into account, defining low-level positive values higher but ≤3 times the upper limit of normal (ULN) and high-level positive >3 times the ULN [27]. However, van der Linden et al. demonstrated that the presence of ACPA, irrespective of their titer, had a better balance between positive and negative likelihood ratios (LR) and between positive and negative predictive values (PPV and NPV, respectively) for RA development in 972 patients with undifferentiated arthritis (UA) [28]. These observations fit with the more recent data from Ten Brinck et al., who reported that ACPA levels were not associated with higher hazards for progression to clinical arthritis in 241 patients with clinically suspect arthralgia [29]. Furthermore, the additive value of ACPA assessment after testing for RF level was higher than vice versa [28], and therefore, the authors proposed updating the 2010 RA criteria by including only ACPA and not assigning different weights to different titers.

Antibodies against citrullinated keratin and filaggrin (AKA) are still assessed with specific assays for research purposes with a sensitivity of 42%–72% and a specificity of 80%–98% based on the employed epitope [30,31,32]. The identification of novel citrullinated epitopes necessarily led to the commercialization of new ELISA kits and eventually to the investigation of how these tests behave in comparison with the CCP2 test [33,34,35,36,37,38,39,40,41,42,43,44,45,46].

Vimentin is an intermediate filament fundamental for cell and, to date, two ELISA assays have detected auto-Abs against its citrullinated form. The first recognizes anticitrullinated vimentin (anti-Savoie/anti-Sa), while the second is directed against the mutated citrullinated vimentin (anti-MCV), a peptide where an arginine residue is replaced by a glycine residue [33].

Anti-Sa auto-Abs display a fairly low sensitivity (37%–50%) but a good specificity (97%–99%) while anti-MCV display a wide range of values for each (39%–78.6% and 74%–100%, respectively), which does not allow judging its performance with reasonable certainty [30,31,47,48,49]. The coexistence of anti-MCV in the majority of patients with anti-CCP confirms that the assessment of the anti-MCV test in this cohort does not provide any additional diagnostic value [44,47,48,49,50,51]. However, the presence of anti-MCV auto-Abs in patients with anti-CCP negative UA is highly predictive of RA development [50].

The immunodominant citrullinated alpha enolase peptide-1 (CEP-1) was identified in RA synovium and SF in 2008 [52] and associated with specific auto-Abs isolated from RA joints [53]. Alpha-enolase 1 is a glycolytic isoenzyme that catalyzes the conversion of 2-phosphoglycerate to phosphoenolpyruvate. Anti-CEP-1 auto-Abs behave similarly to anti-MCV concerning the coexistence with anti-CCP, so the large majority of RA patients are double positive [46]. In striking contrast, because only a very small proportion of patients with UA (<5%) displays anti-CEP-1 antibodies, their usefulness in predicting RA development is negligible [54,55]. Glucose-6-phosphate isomerase (GPI) is another glycolytic enzyme, and antibodies against citrullinated-GPI, targeting different epitopes, have been described in RA [56]. However, when compared to anti-CCP, anti-MCV and anti-AKA, they display a poor specificity (<70%) [30]. Citrullinated fibrin is one of the most relevant autoantigens in the synovial tissue of RA patients [57,58]. The ELISA test assessing anticitrullinated fibrinogen (hFibA) displays a sensitivity ranging between 48% and 73%, with a specificity up to 95% in established RA [59,60] and a sensitivity of 32% with a specificity of 88% in predicting the progression to RA in UA [50]. Over the years, antibodies against a variety of citrullinated collagen epitopes have been described in patients with RA, showing a sensitivity of around 40% and a specificity of around 95% [31,61]. Interestingly, Abs against citrullinated type I and II collagen telopeptides also display a predictive role, being observed in samples of RA patients for years before the onset of the disease [62]. It is worth mentioning that also some isoforms of PADs, the enzymes catalyzing citrullination, may represent a target antigen for auto-Abs in RA [63,64]. Anti-PAD4 Abs are mainly present in anti-CCP positive patients and display a sensitivity ranging from 24% to 37% and a specificity of 95%–100% [47,56,65,66,67], while anti-PAD3 display a very poor sensitivity with a specificity of around 88% [56,67,68]. To note, a subset of anti-PAD3 antibodies cross-reacts with anti-PAD4, and this has been associated with a specific clinical picture as outlined in the next paragraph.

Conflicting results have been reported regarding the actual relevance of detecting multiple ACPA specificities instead of the current gold standard (anti-CCP only) [30,31,47,48,49,50] for diagnostic purposes in the suspicion of RA or to stratify RA patients. As far as the diagnostic aspect is concerned, most ACPA are detectable only in anti-CCP positive subjects; hence, additional costs to gather the same information are not justifiable in routine clinical practice. The identification of ACPA specificities that are present also in patients that do not display anti-CCP or RF but fulfill the other criteria for RA may open a new scenario and put the basis to reconsider the current approach (Table 1).

With regard to subtyping RA patients, the question remains on how useful ACPA specificities can be to identify patients at higher risk of more severe disease (namely, erosive disease or extra-articular manifestation) and tailor the follow-up schedule and follow-up strategy accordingly. The next paragraph summarizes the available evidence in this field.

### 2.2. ACPA and Disease Activity, Bone Damage, and Extra-Articular Manifestations

Although the diagnostic role of ACPA, namely, anti-CCP, is well established, their pathogenic role in RA remains a matter of intense debate and investigation, since proof of pathogenicity requires both direct and indirect evidence that an autoimmune response causes pathology [69]. Given the cross-reactivity of ACPA and their capability to bind a large number of citrullinated proteins, along with the fact that the exact location of target antigens in RA is largely unknown, and the limitations of animal models of arthritis, we are still far from solid conclusions in this regard [70]. Interestingly, several studies demonstrated that in patients with a double positivity for both RF and anti-CCP, the disease is generally more severe, with a poorer prognosis, and therefore requiring more aggressive treatment [28,71]. However, if the relationship between RF and disease activity is well established, this is not confirmed for ACPA, outlining the so-called ‘ACPA paradox’. Therefore, while the paradigmatic belief that disease activity causes structural progression overtime in RA underpins the prognostic role of chronic inflammation, and therefore of RF as one of its triggers, the association of anti-CCP and progression of structural joint damage puts forward the hypothesis that seropositive disease in itself confers an independent risk for disease progression [72]. Nonetheless, other specific ACPAs seem to be associated with chronic inflammation and disease activity [47,49,50], but their association with erosive disease is unclear. This is the case, for instance, of anti-Sa that are correlated to disease activity [47,49] but data concerning the association with radiological articular damage are conflicting [48,49,61], or anti-MCV that are correlated to disease activity, erythrocyte sedimentation rate (ESR), and C reactive protein (CRP), pro-inflammatory cytokine concentration, and erosive disease [44,47,49]. The same applies to anti-CEP-1 that were found associated with erosions in most [45,54,73] but not all [74] available studies. Furthermore, according to Alunno et al., this association was stronger in anti-CEP1 positive/anti-CCP2 negative compared to double positive patients [45]. As far as anti-PAD antibodies are concerned, both those targeting only the isoform 4 [75,76,77] and those that cross-react with isoforms 3 and 4 [68] have been linked to erosive disease. However, the latter seem to be a stronger determinant of erosive disease since the total Sharp van der Heidje score (SvdH) at baseline is higher in patients with anti-PAD3 cross-reactive antibodies not only compared to anti-PAD negative patients but also to anti-PAD4 positive patients [68]. Of interest, this association was independent of other predictors of erosive disease, such as anti-CCP positivity or presence of shared-epitope alleles.

Interstitial lung disease (ILD) is one of the most relevant extra-articular manifestations in terms of burden, with a prevalence ranging from 4 to 70% [78] and impact on disease prognosis [79]. The spectrum of RA-associated ILD encompasses mild reversible inflammatory conditions and severe rapidly progressing fibrotic diseases that represent one of the most common causes of death in RA patients, hence requiring prompt recognition and treatment [80]. RF positivity was identified as a trigger for ILD nearly 50 years ago [81], and more recently, anti-CCP have also been associated with ILD [82]. Two recent studies demonstrated that anti-CEP1 antibodies are also associated with ILD [45,73] and, interestingly, in the article by Alunno et al., the prevalence of RA-associated ILD was higher in patients with single positivity for anti-CEP-1 compared to those with single positivity for anti-CCP. The question remains on whether assessment of anti-CEP1 at the time of RA diagnosis may have a predictive role for occurrence and timing of ILD and therefore allow identifying patients at risk in order to start a thorough follow-up. Anti-PAD3/4 cross-reactive antibodies have also been associated with ILD [67]. Finally, with regard to other extra-articular manifestations, such as cutaneous vasculitis and rheumatoid nodules, an association with anti-MCV has been reported [83,84] (Table 1).

## 3. Antibodies against Modified Proteins (AMPA)

### 3.1. Antibodies Anticarbamylated Proteins

Carbamylation is a chemical PTM induced by the presence of cyanate in which the enzyme lysine carbamyltransferase catalyzes the carbamylation of a lysine residue into homocitrulline. In normal conditions, cyanate levels are too low to induce consistent carbamylation, but conditions like uremia, inflammation, and the exposure to cigarette smoke enhance cyanate levels [85,86]. Auto-Abs targeting carbamylated proteins (CarP), in particular, carbamylated fetal calf serum (Ca-FCS), carbamylated fibrinogen (Ca-Fib) and carbamylated vimentin (CarbVim), have been identified in the sera of RA patients [87,88]. According to a recent literature review, prevalence of anti-Ca-FCS in RA ranges from 34% to 53% [89], while the prevalence of anti-CarbVim in RA is 48% [88]. Considering all anti-CarP Abs, the sensitivity is 18%–26% in patients prior to the diagnosis, and 27%–46% in patients with overt RA, while the specificity varies between 89% and 97% [51,90]. Anti-CarP are often associated with anti-CCP and anti-Sa, and the titers of the three are directly correlated [51], while only 6%–30% of seronegative RA patients are positive for anti-CarP (6–30%) [91]. Although only a small proportion of seronegative RA patients are positive for anti-CarP prior to diagnosis (11%), the presence of anti-CarP in patients with arthralgia or UA is a strong predictor of RA development regardless of the ACPA/RF status [92,93]. Interestingly, the presence of anti-CarP before disease onset is also a prognostic factor with regard to erosive disease [90]. However, data regarding the correlation of anti-CarP and disease activity or erosive disease in patients with established RA are conflicting, although the majority of studies agree that they are associated with radiological damage [51,90,93,94] (Table 1).

### 3.2. Antibodies Antiacetylated Proteins

As outlined above, vimentin can undergo citrullination and carbamylation, thereby inducing an aberrant autoimmune response in RA. It was therefore investigated whether this protein could undergo other PTMs, and in 2016, Juarez et al. provided evidence of antiacetylated vimentin (AcetVim) antibodies in early RA [95]. It is interesting to note that while in patients with established RA, the proportion of patients with anti-AcetVim IgG is around 35% and significantly higher compared to patients with persistent non-RA arthritis, the prevalence anti-AcetVim IgA is absolutely overlapping in these two groups and is again about 35%. This leads to a consistent gap in terms of specificity which is 86.2% for IgG and only 64.7% for IgA.

When considering the co-expression of anti-AcetVim IgG or IgA according to the anti-CCP status, it can be noted that anti-CCP negative patients behave similarly to persistent non-RA arthritis, with a prevalence of IgG significantly lower compared to anti-CCP positive patients [95]. Likewise, no difference in the prevalence of anti-AcetVim IgA can be observed between anti-CCP positive and anti-CCP negative patients. Although these are the first demonstration of such reactivity in RA, additional studies investigating other acetylated targets are needed to identify auto-Abs with a diagnostic relevance in seronegative RA (Table 1).

## 4. Conclusions and Future Perspectives

In the late 1990s, the discovery that citrullinated proteins are targeted by auto-Abs, called ACPA, revolutionized the field of research in RA. Citrullination is one out of many post-translational modifications (PTMs) of proteins, and over the last decades, an increasing number of studies revealed that a wide number of peptides could undergo PTMs in predisposed patients, including several other PTMs such as carbamylation and acetylation, leading to the development of novel epitopes and eventually auto-Abs against them. Conflicting results have been reported regarding the actual relevance of detecting multiple ACPA specificities instead of the current gold standard (anti-CCP2 only) in the suspicion of RA. Moreover, additional studies are required to evaluate the utility of ACPA and AMPA specificities in stratifying RA patients at the time of diagnosis, to tailor the follow-up schedule and follow-up strategy. In this regard, it is compelling that unlike in the past, every study reports sensitivity and specificity of the assay rather than only the prevalence of novel auto-autoantibodies in order to allow a proper comparison with anti-CCP. Finally, the search must continue to determine whether biomarkers ACPA and AMPA have a direct pathogenic role in arthritis or whether these antibodies more simply reflect underlying T- and B-cell responses without directly impacting on joint inflammation and damage.

## Figures and Tables

**Table 1:** Role of antibodies against post-translationally modified proteins in rheumatoid arthritis.

					Associations				
Antibody	RA Prediction	Se%	Sp%	RA Classification Criteria	Disease Activity and/or Inflammatory Status	Erosive Disease	ILD	Rheumatoid Vasculitis	Ref
Anti-CCP	Y	68	98	Y	N	Y	Y	Y	[14,24,25,27,28,29,33,34,35,36,37,38,39,40,41,42,43,44,45,46,71,72,82]
Anti-Sa	N	37–50	97–99	N	Y	Y/N	nd	nd	[30,31,47,48,49,61]
Anti-MCV	Y	39–78.6	74–100	N	Y	Y	nd	Y	[30,31,44,47,48,49,50,51,83,84]
Anti-CEP-1	N	nd	nd	N	N	Y	Y	nd	[45,53,54,55,73,74]
Anti-GPI	N	75	64.3	N	nd	nd	nd	nd	[30,56]
Anti-hFibA	Y	48–73	95	N	nd	nd	nd	nd	[50,59,60]
Anti-CitCol I and II	Y	41–47	94–96	N	nd	nd	nd	nd	[31,61,62]
Anti-PAD3	N	nd	88	N	N	N	nd	nd	[56,67,68]
Anti-PAD4	N	24–37	95–100	N	N	Y	nd	nd	[47,56,65,66,67,75,76,77]
Anti-PAD3/4	N	nd	nd	N	N	Y	Y	nd	[67,68]
Anti-CarP	Y	18–26	89–97	N	Y/N	Y	nd	nd	[51,88,89,90,91,92,93,94]
Anti-AcetVim	N	33.7–66.3	65.6–88.6	N	nd	nd	nd	nd	[95]

RA, rheumatoid arthritis; Se, sensitivity; Sp, specificity; ILD, interstitial lung disease; Y, yes; N, no; nd, no data reported; CCP, cyclic citrullinated peptide; MCV, mutated citrullinated vimentin; CEP, citrullinated alpha enolase peptide; GPI, glucose-6-phosphate isomerase; hFIbA, citrullinated fibrinogen; CitCol, citrullinated collagen; PAD, peptidylarginine deiminase; CarP, carbamylated proteins; AcetVim, acetylated vimentin.

## References

[1] Smolen J.S., Aletaha D., Barton A., Burmester G.R., Emery P., Firestein G., Kavanaugh A., McInnes I.B., Solomon D.H., Strand V. (2018). Rheumatoid arthritis. Nat. Rev. Dis. Primers..

[2] Marcucci E., Bartoloni E., Alunno A., Leone M. C., Cafaro G., Luccioli F., Valentini V., Valentini E., La Paglia G.M.C., Bonifacio A.F. (2018). Extra-articular rheumatoid arthritis. Reumatismo.

[3] Pike R.M., Sulkin S.E., Coggeshall H.C. (1949). Serological reactions in rheumatoid arthritis; factors affecting the agglutination of sensitized sheep erythrocytes in rheumatid-arthritis serum. J. Immunol..

[4] Carubbi F., Alunno A., Cipriani P., Bistoni O., Scipioni R., Liakouli V., Ruscitti P., Berardicurti O., Di Bartolomeo S., Gerli R. (2019). Laboratory Assessment of Patients with Suspected Rheumatic Musculoskeletal Diseases: Challenges and Pitfalls. Curr. Rheumatol. Rev..

[5] Sun J., Zhang Y., Liu L., Liu G. (2014). Diagnostic accuracy of combined tests of anti cyclic citrullinated peptide antibody and rheumatoid factor for rheumatoid arthritis: A meta-analysis. Clin. Exp. Rheumatol..

[6] Nienhuis R.L., Mandema E. (1964). A new serum factor in patients with rheumatoid arthritis; the antiperinuclear factor. Ann. Rheum. Dis..

[7] Hoet R.M., Boerbooms A.M., Arends M., Ruiter D.J., van Venrooij W.J. (1991). Antiperinuclear factor, a marker autoantibody for rheumatoid arthritis: Colocalisation of the perinuclear factor and profilaggrin. Ann. Rheum. Dis..

[8] Hoet R.M., Voorsmith R.A., van Venrooij W.J. (1991). The perinuclear factor, a rheumatoid arthritis-specific autoantigen, is not present in keratohyalin granules of cultured buccal mucosa cells. Clin. Exp. Immunol..

[9] Baka Z., György B., Géher P., Buzás E.I., Falus A., Nagy G. (2012). Citrullination under physiological and pathological conditions. Joint Bone Spine.

[10] Koziel J., Mydel P., Potempa J. (2014). The link between periodontal disease and rheumatoid arthritis: An updated review. Curr Rheumatol Rep.

[11] Olsen I., Singhrao S.K., Potempa J. (2018). Citrullination as a plausible link to periodontitis, rheumatoid arthritis, atherosclerosis and Alzheimer’s disease. J Oral Microbiol.

[12] Tilvawala R., Nguyen S.H., Maurais A.J., Nemmara V.V., Nagar M., Salinger A.J., Nagpal S., Weerapana E., Thompson P.R. (2018). The Rheumatoid Arthritis-Associated Citrullinome. Cell. Chem. Biol..

[13] Gasparyan A.Y., Ayvazyan L., Blackmore H., Kitas G.D. (2011). Writing a narrative biomedical review: Considerations for authors, peer reviewers, and editors. Rheumatol. Int..

[14] van Venrooij W.J., van Beers J.J., Pruijn G.J. (2011). Anti-CCP antibodies: The past, the present and the future. Nat. Rev. Rheumatol..

[15] Suzuki A., Yamada R., Chang X., Tokuhiro S., Sawada T., Suzuki M., Nagasaki M., Nakayama-Hamada M., Kawaida R., Ono M. (2003). Functional haplotypes of PADI4, encoding citrullinating enzyme peptidylarginine deiminase 4, are associated with rheumatoid arthritis. Nat Genet.

[16] Valesini G., Gerardi M.C., Iannuccelli C., Pacucci V.A., Pendolino M., Shoenfeld Y. (2015). Citrullination and autoimmunity. Autoimmun. Rev..

[17] Ioan-Facsinay A., el-Bannoudi H., Scherer H.U., van der Woude D., Ménard H.A., Lora M., Trouw L.A., Huizinga T.W., Toes R.E. (2011). Anti-cyclic citrullinated peptide antibodies are a collection of anti-citrullinated protein antibodies and contain overlapping and non-overlapping reactivities. Ann. Rheum. Dis..

[18] Masson-Bessière C., Sebbag M., Girbal-Neuhauser E., Nogueira L., Vincent C., Senshu T., Serre G. (2001). The major synovial targets of the rheumatoid arthritis-specific antifilaggrin autoantibodies are deiminated forms of the alpha- and beta-chains of fibrin. J. Immunol..

[19] Khandpur R., Carmona-Rivera C., Vivekanandan-Giri A., Gizinski A., Yalavarthi S., Knight J.S., Friday S., Li S., Patel R.M., Subramanian V. (2013). NETs are a source of citrullinated autoantigens and stimulate inflammatory responses in rheumatoid arthritis. Sci Transl Med.

[20] Chapuy-Regaud S., Sebbag M., Baeten D., Clavel C., Foulquier C., De Keyser F., Serre G. (2005). Fibrin deimination in synovial tissue is not specific for rheumatoid arthritis but commonly occurs during synovitides. J. Immunol..

[21] Gabarrini G., de Smit M., Westra J., Brouwer E., Vissink A., Zhou K., Rossen J.W., Stobernack T., van Dijl J.M., van Winkelhoff A.J. (2015). The peptidylarginine deiminase gene is a conserved feature of Porphyromonas gingivalis. Sci. Rep..

[22] Altman D., Bland J. (1994). Statistics Notes: Diagnostic tests 1: Sensitivity and specificity. BMJ.

[23] Cardoso J.R., Pereira L.M., Iversen M.D., Ramos A.L. (2014). What is gold standard and what is ground truth?. Dental Press J Orthod.

[24] Schellekens G.A., de Jong B.A., van den Hoogen F.H., van de Putte L.B., van Venrooij W.J. (1998). Citrulline is an essential constituent of antigenic determinants recognized by rheumatoid arthritis-specific autoantibodies. J. Clin. Investig..

[25] Schellekens G.A., Visser H., de Jong B.A., van den Hoogen F.H., Hazes J.M., Breedveld F.C., van Venrooij W.J. (2000). The diagnostic properties of rheumatoid arthritis antibodies recognizing a cyclic citrullinated peptide. Arthritis Rheum..

[26] Aletaha D., Neogi T., Silman A.J., Funovits J., Felson D.T., Bingham C.O., Birnbaum N.S., Burmester G.R., Bykerk V.P., Cohen M.D. (2010). 2010 Rheumatoid arthritis classification criteria: An American College of Rheumatology/European League Against Rheumatism collaborative initiative. Arthritis Rheum..

[27] van Heemst J., Trouw L.A., Nogueira L., van Steenbergen H.W., van der Helm-van A.H., Allaart C.F., Serre G., Holmdahl R., Huizinga T.W., Toes R.E. (2015). An investigation of the added value of an ACPA multiplex assay in an early rheumatoid arthritis setting. Arthritis Res. Ther..

[28] van der Linden M.P., Batstra M.R., Bakker-Jonges L.E., Detert J., Bastian H., Scherer H.U., Toes R.E., Burmester G.R., Mjaavatten M.D., Kvien T.K. (2011). Toward a data-driven evaluation of the 2010 American College of Rheumatology/European League Against Rheumatism criteria for rheumatoid arthritis: Is it sensible to look at levels of rheumatoid factor?. Arthritis Rheum..

[29] Ten Brinck R.M., van Steenbergen H.W., van Delft M.A.M., Verheul M.K., Toes R.E.M., Trouw L.A., van der Helm-van Mil A.H.M. (2017). The risk of individual autoantibodies, autoantibody combinations and levels for arthritis development in clinically suspect arthralgia. Rheumatology (Oxf.).

[30] Zhu T., Feng L. (2013). Comparison of anti-mutated citrullinated vimentin, anti-cyclic citrullinated peptides, anti-glucose-6-phosphate isomerase and anti-keratin antibodies and rheumatoid factor in the diagnosis of rheumatoid arthritis in Chinese patients. Int. J. Rheum. Dis..

[31] Szarka E., Babos F., Magyar A., Huber K., Szittner Z., Papp K., Prechl J., Pozsgay J., Neer Z., Ádori M. (2014). Recognition of new citrulline-containing peptide epitopes by autoantibodies produced in vivo and in vitro by B cells of rheumatoid arthritis patients. Immunology.

[32] Wang X., Chen P., Cui J., Yang C., Du H. (2015). Keratin 8 is a novel autoantigen of rheumatoid arthritis. Biochem. Biophys. Res. Commun..

[33] Bang H., Egerer K., Gauliard A., Lüthke K., Rudolph P.E., Fredenhagen G., Berg W., Feist E., Burmester G.R. (2007). Mutation and citrullination modifies vimentin to a novel autoantigen for rheumatoid arthritis. Arthritis Rheum..

[34] Mathsson L., Mullazehi M., Wick M.C., Sjöberg O., van Vollenhoven R., Klareskog L., Rönnelid J. (2008). Antibodies against citrullinated vimentin in rheumatoid arthritis: Higher sensitivity and extended prognostic value concerning future radiographic progression as compared with antibodies against cyclic citrullinated peptides. Arthritis Rheum..

[35] Vander Cruyssen B., Cantaert T., Nogueira L., Clavel C., De Rycke L., Dendoven A., Sebag M., Deforce D., Vincent C., Elewaut D. (2006). Diagnostic value of anti-human citrullinated fibrinogen ELISA and comparison with four other anti-citrullinated protein assays. Arthritis Res. Ther..

[36] Bizzaro N., Tonutti E., Tozzoli R., Villalta D. (2007). Analytical and diagnostic characteristics of 11 2nd- and 3rd-generation immunoenzymatic methods for the detection of antibodies to citrullinated proteins. Clin. Chem..

[37] Coenen D., Verschueren P., Westhovens R., Bossuyt X. (2007). Technical and diagnostic performance of 6 assays for the measurement of citrullinated protein/peptide antibodies in the diagnosis of rheumatoid arthritis. Clin. Chem..

[38] Dejaco C., Klotz W., Larcher H., Duftner C., Schirmer M., Herold M. (2006). Diagnostic value of antibodies against a modified citrullinated vimentin in rheumatoid arthritis. Arthritis Res. Ther..

[39] Mutlu N., Bicakcigil M., Tasan D.A., Kaya A., Yavuz S., Ozden A.I. (2009). Comparative performance analysis of 4 different anti-citrullinated protein assays in the diagnosis of rheumatoid arthritis. J. Rheumatol..

[40] Damjanovska L., Thabet M.M., Levarth E.W., Stoeken-Rijsbergen G., van der Voort E.I., Toes R.E., Huizinga T.W., van der Helm-van Mil A.H. (2010). Diagnostic value of anti-MCV antibodies in differentiating early inflammatory arthritis. Ann. Rheum. Dis..

[41] Innala L., Kokkonen H., Eriksson C., Jidell E., Berglin E., Dahlqvst S.R. (2008). Antibodies against mutated citrullinated vimentin are a better predictor of disease activity at 24 months in early rheumatoid arthritis than antibodies against cyclic citrullinated peptides. J. Rheumatol..

[42] Soós L., Szekanecz Z., Szabó Z., Fekete A., Zeher M., Horváth I.F., Dankó K., Kapitány A., Végvári A., Sipka S. (2007). Clinical evaluation of anti-mutated citrullinated vimentin by ELISA in rheumatoid arthritis. J. Rheumatol..

[43] Vander Cruyssen B., Nogueira L., Van Praet J., Deforce D., Elewaut D., Serre G., De Keyser F. (2008). Do all anti-citrullinated protein/peptide antibody tests measure the same? Evaluation of discrepancy between anti-citrullinated protein/peptide antibody tests in patients with and without rheumatoid arthritis. Ann. Rheum. Dis..

[44] Bartoloni E., Alunno A., Bistoni O., Bizzaro N., Migliorini P., Morozzi G., Doria A., Mathieu A., Lotzniker M., Allegri F. (2012). Diagnostic value of anti-mutated citrullinated vimentin in comparison to anti-cyclic citrullinated peptide and anti-viral citrullinated peptide 2 antibodies in rheumatoid arthritis: An Italian multicentric study and review of the literature. Autoimmun. Rev..

[45] Alunno A., Bistoni O., Pratesi F., La Paglia G.M.C., Puxeddu I., Migliorini P., Gerli R. (2018). Anti-citrullinated alpha enolase antibodies, interstitial lung disease and bone erosion in rheumatoid arthritis. Rheumatology (Oxf.).

[46] Manca M.L., Alunno A., D’Amato C., Bistoni O., Puxeddu I., Gerli R., Migliorini P., Pratesi F. (2018). Anti -citrullinated peptide antibodies profiling in established rheumatoid arthritis. Joint Bone Spine..

[47] Reyes-Castillo Z., Palafox-Sánchez C.A., Parra-Rojas I., Martínez-Bonilla G.E., del Toro-Arreola S., Ramírez-Dueñas M.G., Ocampo-Bermudes G., Muñoz-Valle J.F. (2015). Comparative analysis of autoantibodies targeting peptidylarginine deiminase type 4, mutated citrullinated vimentin and cyclic citrullinated peptides in rheumatoid arthritis: Associations with cytokine profiles, clinical and genetic features. Clin. Exp. Immunol..

[48] Iwaszkiewicz C., Puszczewicz M., Białkowska-Puszczewicz G. (2015). Diagnostic value of the anti-Sa antibody compared with the anti-cyclic citrullinated peptide antibody in rheumatoid arthritis. Int. J. Rheum. Dis..

[49] Hou Y.F., Sun G.Z., Sun H.S., Pan W.P., Liu W.B., Zhang C.Q. (2012). Diagnostic value of anti-Sa and anticitrullinated protein antibodies in rheumatoid arthritis. J. Rheumatol..

[50] Nicaise-Roland P., Nogueira L., Demattei C., de Chaisemartin L., Rincheval N., Cornillet M., Grootenboer-Mignot S., Dieudé P., Dougados M., Cantagrel A. (2013). Autoantibodies to citrullinated fibrinogen compared with anti-MCV and anti-CCP2 antibodies in diagnosing rheumatoid arthritis at an early stage: Data from the French ESPOIR cohort. Ann. Rheum. Dis..

[51] Challener G.J., Jones J.D., Pelzek A.J., Hamilton B.J., Boire G., de Brum-Fernandes A.J., Masetto A., Carrier N., Ménard H.A., Silverman G.J. (2016). Anti-carbamylated Protein Antibody Levels Correlate with Anti-Sa (Citrullinated Vimentin) Antibody Levels in Rheumatoid Arthritis. J. Rheumatol..

[52] Kinloch A., Lundberg K., Wait R., Wegner N., Lim N.H., Zendman A.J., Saxne T., Malmström V., Venables P.J. (2008). Synovial fluid is a site of citrullination of autoantigens in inflammatory arthritis. Arthritis Rheum..

[53] Snir O., Widhe M., Hermansson M., von Spee C., Lindberg J., Hensen S., Lundberg K., Engström A., Venables P.J., Toes R.E. (2010). Antibodies to several citrullinated antigens are enriched in the joints of rheumatoid arthritis patients. Arthritis Rheum..

[54] Montes A., Dieguez-Gonzalez R., Perez-Pampin E., Calaza M., Mera-Varela A., Gomez-Reino J.J., Gonzalez A. (2011). Particular association of clinical and genetic features with autoimmunity to citrullinated α-enolase in rheumatoid arthritis. Arthritis Rheum..

[55] Ponikowska M., Świerkot J., Nowak B., Korman L., Wiland P. (2018). Anti-cep-1 antibodies and other autoantibodies in early arthritis. Ann. Rheum. Dis..

[56] Umeda N., Matsumoto I., Ito I., Kawasaki A., Tanaka Y., Inoue A., Tsuboi H., Suzuki T., Hayashi T., Ito S. (2013). Anti-citrullinated glucose-6-phosphate isomerase peptide antibodies in patients with rheumatoid arthritis are associated with HLA-DRB1 shared epitope alleles and disease activity. Clin. Exp. Immunol..

[57] Sebbag M., Monard N., Auger I., Clavel C., Arnaud J., Nogueira L., Roudier J., Serre G. (2006). Epitopes of human fibrin recognized by the rheumatoid arthritis-specific autoantibodies to citrullinated proteins. Eur. J. Immunol..

[58] Nogueira N.L., Sebbag M., Chapuy-Regaud S., Clavel C., Fournie B., Cantagrel A., Vincent C., Serre G. (2002). Autoantibodies to deiminated fibrinogen are the most efficient serological criterion for the diagnosis of rheumatoid arthritis. Arthritis Res. Ther..

[59] Cornillet M., Sebbag M., Verrouil E., Magyar A., Babos F., Ruyssen-Witrand A., Hudecz F., Cantagrel A., Serre G., Nogueira L. (2014). The fibrin-derived citrullinated peptide β60-74Cit_60,72,74_ bears the major ACPA epitope recognised by the rheumatoid arthritis-specific anticitrullinated fibrinogen autoantibodies and anti-CCP2 antibodies. Ann. Rheum. Dis..

[60] Nogueira L., Cornillet M., Singwe-Ngandeu M., Viatte S., Bas S., Gabay C., Serre G. (2014). In Black Africans with rheumatoid arthritis, ACPA recognize citrullinated fibrinogen and the derived peptides α36-50Cit38,42 and β60-74Cit60,72,74, like in Caucasians. Clin. Immunol..

[61] Turunen S., Hannonen P., Koivula M.K., Risteli L., Risteli J. (2015). Separate and overlapping specificities in rheumatoid arthritis antibodies binding to citrulline- and homocitrulline-containing peptides related to type I and II collagen telopeptides. Arthritis Res. Ther..

[62] Koivula M.K., Heliövaara M., Rissanen H., Palosuo T., Knekt P., Immonen H., Risteli J. (2012). Antibodies binding to citrullinated telopeptides of type I and type II collagens and to mutated citrullinated vimentin synergistically predict the development of seropositive rheumatoid arthritis. Ann. Rheum. Dis..

[63] Foulquier C., Sebbag M., Clavel C., Chapuy-Regaud S., Al Badine R., Méchin M.C., Vincent C., Nachat R., Yamada M., Takahara H. (2007). Peptidyl arginine deiminase type 2 (PAD-2) and PAD-4 but not PAD-1, PAD-3, and PAD-6 are expressed in rheumatoid arthritis synovium in close association with tissue inflammation. Arthritis Rheum..

[64] Halvorsen E.H., Pollmann S., Gilboe I.M., van der Heijde D., Landewé R., Ødegård S., Kvien T.K., Molberg Ø. (2008). Serum IgG antibodies to peptidylarginine deiminase 4 in rheumatoid arthritis and associations with disease severity. Ann. Rheum. Dis..

[65] Ishigami A., Uchida Y., Miyazaki T., Handa S., Choi E.K., Kim Y.S., Kasahara Y., Maruyama N. (2013). Two novel sandwich ELISAs identify PAD4 levels and PAD4 autoantibodies in patients with rheumatoid arthritis. Mod. Rheumatol..

[66] Ferucci E.D., Darrah E., Smolik I., Choromanski T.L., Robinson D.B., Newkirk M.M., Fritzler M.J., Rosen A., El-Gabalawy H.S. (2013). Prevalence of anti-peptidylarginine deiminase type 4 antibodies in rheumatoid arthritis and unaffected first-degree relatives in indigenous North American Populations. J. Rheumatol..

[67] Giles J.T., Darrah E., Danoff S., Johnson C., Andrade F., Rosen A., Bathon J.M. (2014). Association of cross-reactive antibodies targeting peptidyl-arginine deiminase 3 and 4 with rheumatoid arthritis-associated interstitial lung disease. PLoS ONE.

[68] Darrah E., Giles J.T., Ols M.L., Bull H.G., Andrade F., Rosen A. (2013). Erosive rheumatoid arthritis is associated with antibodies that activate PAD4 by increasing calcium sensitivity. Sci. Transl. Med..

[69] Rose N.R., Bona C. (1993). Defining criteria for autoimmune diseases (Witebsky’s postulates revisited). Immunol Today.

[70] Toes R., Pisetsky D.S. (2019). Pathogenic effector functions of ACPA: Where do we stand?. Ann. Rheum. Dis..

[71] Jilani A.A., Mackworth-Young C.G. (2015). The role of citrullinated protein antibodies in predicting erosive disease in rheumatoid arthritis: A systematic literature review and meta-analysis. Int. J. Rheumatol..

[72] Aletaha D., Blüml S. (2016). Therapeutic implications of autoantibodies in rheumatoid arthritis. RMD Open.

[73] Liu Y., Liu C., Li L., Zhang F., Li Y., Zhang S. (2019). High levels of antibodies to citrullinated α-enolase peptide-1 (CEP-1) identify erosions and interstitial lung disease (ILD) in a Chinese rheumatoid arthritis cohort. Clin. Immunol..

[74] Fisher B.A., Plant D., Brode M., van Vollenhoven R.F., Mathsson L., Symmons D., Lundberg K., Rönnelid J., Venables P.J. (2011). Antibodies to citrullinated α-enolase peptide 1 and clinical and radiological outcomes in rheumatoid arthritis. Ann. Rheum. Dis..

[75] Harris M.L., Darrah E., Lam G.K., Bartlett S.J., Giles J.T., Grant A.V., Gao P., Scott W.W., El-Gabalawy H., Casciola-Rosen L. (2008). Association of autoimmunity to peptidyl arginine deiminase type 4 with genotype and disease severity in rheumatoid arthritis. Arthritis Rheum..

[76] Kolfenbach J.R., Deane K.D., Derber L.A., O’Donnell C.I., Gilliland W.R., Edison J.D., Rosen A., Darrah E., Norris J.M., Holers V.M. (2010). Autoimmunity to peptidyl arginine deiminase type 4 precedes clinical onset of rheumatoid arthritis. Arthritis Rheum..

[77] Halvorsen E.H., Haavardsholm E.A., Pollmann S., Boonen A., van der Heijde D., Kvien T.K., Molberg Ø. (2009). Serum IgG antibodies to peptidylarginine deiminase 4 predict radiographic progression in patients with rheumatoid arthritis treated with tumour necrosis factor-alpha blocking agents. Ann. Rheum. Dis..

[78] Wallace B., Vummidi D., Khanna D. (2016). Management of connective tissue diseases associated interstitial lung disease: A review of the published literature. Curr. Opin. Rheumatol..

[79] Alunno A., Gerli R., Giacomelli R., Carubbi F. (2017). Clinical, Epidemiological, and Histopathological Features of Respiratory Involvement in Rheumatoid Arthritis. Biomed. Res. Int..

[80] Pinheiro F.A., Souza D.C., Sato E.I. (2015). A Study of Multiple Causes of Death in Rheumatoid Arthritis. J. Rheumatol..

[81] DeHoratius R.J., Williams R.C. (1972). Rheumatoid factor accentuation of pulmonary lesions associated with experimental diffuse proliferative lung disease. Arthritis Rheum..

[82] Kelly C.A., Saravanan V., Nisar M., Arthanari S., Woodhead F.A., Price-Forbes A.N., Dawson J., Sathi N., Ahmad Y., Koduri G. (2014). Rheumatoid arthritis-related interstitial lung disease: Associations, prognostic factors and physiological and radiological characteristics--a large multicentre UK study. Rheumatology (Oxf.).

[83] Turesson C., Mathsson L., Jacobsson L.T., Sturfelt G., Rönnelid J. (2013). Antibodies to modified citrullinated vimentin are associated with severe extra-articular manifestations in rheumatoid arthritis. Ann. Rheum. Dis..

[84] Gonzalez-Lopez L., Rocha-Muñoz A.D., Ponce-Guarneros M., Flores-Chavez A., Salazar-Paramo M., Nava A., Cardona-Muñoz E.G., Fajardo-Robledo N.S., Zavaleta-Muñiz S.A., Garcia-Cobian T. (2014). Anti-cyclic citrullinated peptide (anti-CCP) and anti-mutated citrullinated vimentin (anti-MCV) relation with extra-articular manifestations in rheumatoid arthritis. J. Immunol. Res..

[85] Pruijn G.J. (2015). Citrullination and carbamylation in the pathophysiology of rheumatoid arthritis. Front. Immunol..

[86] Wang Z., Nicholls S.J., Rodriguez E.R., Kummu O., Hörkkö S., Barnard J., Reynolds W.F., Topol E.J., DiDonato J.A., Hazen S.L. (2007). Protein carbamylation links inflammation, smoking, uremia and atherogenesis. Nat. Med..

[87] Shi J., van Veelen P.A., Mahler M., Janssen G.M., Drijfhout J.W., Huizinga T.W., Toes R.E., Trouw L.A. (2014). Carbamylation and antibodies against carbamylated proteins in autoimmunity and other pathologies. Autoimmun. Rev..

[88] Ospelt C., Bang H., Feist E., Camici G., Keller S., Detert J., Krämer A., Gay S., Ghannam K., Burmester G.R. (2017). Carbamylation of vimentin is inducible by smoking and represents an independent autoantigen in rheumatoid arthritis. Ann. Rheum. Dis..

[89] Verheul M.K., Böhringer S., van Delft M.A.M., Jones J.D., Rigby W.F.C., Gan R.W., Holers V.M., Edison J.D., Deane K.D., Janssen K.M.J. (2018). Triple Positivity for Anti-Citrullinated Protein Autoantibodies, Rheumatoid Factor, and Anti-Carbamylated Protein Antibodies Conferring High Specificity for Rheumatoid Arthritis: Implications for Very Early Identification of At-Risk Individuals. Arthritis Rheumatol..

[90] Brink M., Verheul M.K., Rönnelid J., Berglin E., Holmdahl R., Toes R.E., Klareskog L., Trouw L.A., Rantapää-Dahlqvist S. (2015). Anti-carbamylated protein antibodies in the pre-symptomatic phase of rheumatoid arthritis, their relationship with multiple anti-citrulline peptide antibodies and association with radiological damage. Arthritis Res. Ther..

[91] Gan R.W., Trouw L.A., Shi J., Toes R.E., Huizinga T.W., Demoruelle M.K., Kolfenbach J.R., Zerbe G.O., Deane K.D., Edison J.D. (2015). Anti-carbamylated protein antibodies are present prior to rheumatoid arthritis and are associated with its future diagnosis. J. Rheumatol..

[92] Shi J., van Steenbergen H.W., van Nies J.A., Levarht E.W., Huizinga T.W., van der Helm-van Mil A.H., Toes R.E., Trouw L.A. (2015). The specificity of anti-carbamylated protein antibodies for rheumatoid arthritis in a setting of early arthritis. Arthritis Res. Ther..

[93] Humphreys J.H., Verheul M.K., Barton A., MacGregor A.J., Lunt M., Toes R.E., Symmons D.P., Trouw L.A., Verstappen S.M. (2016). Anticarbamylated protein antibodies are associated with long-term disability and increased disease activity in patients with early inflammatory arthritis: Results from the Norfolk Arthritis Register. Ann. Rheum. Dis..

[94] Forslind K., Ahlmén M., Eberhardt K., Hafström I., Svensson B., BARFOT Study Group (2004). Prediction of radiological outcome in early rheumatoid arthritis in clinical practice: Role of antibodies to citrullinated peptides (anti-CCP). Ann. Rheum. Dis..

[95] Juarez M., Bang H., Hammar F., Reimer U., Dyke B., Sahbudin I., Buckley C.D., Fisher B., Filer A., Raza K. (2016). Identification of novel antiacetylated vimentin antibodies in patients with early inflammatory arthritis. Ann. Rheum. Dis..

